# Complete Nucleotide Sequence Analysis of a Novel *Bacillus subtilis*-Infecting Bacteriophage BSP10 and Its Effect on Poly-Gamma-Glutamic Acid Degradation

**DOI:** 10.3390/v10050240

**Published:** 2018-05-04

**Authors:** Kuntal Ghosh, Amal Senevirathne, Hai Seong Kang, Woo Bin Hyun, Ji Eun Kim, Kwang-Pyo Kim

**Affiliations:** Department of Food Science and Technology, College of Agriculture and Life Sciences, Chonbuk National University, Jeonju, Jeollabuk-do 561-756, Korea; micro.kuntal@jbnu.ac.kr (K.G.); amal_senevirathne@yahoo.com (A.S.); rkdgotjd12@jbnu.ac.kr (H.S.K.); mlnkop753@jbnu.ac.kr (W.B.H.); jiun4141@gmail.com (J.E.K.)

**Keywords:** *Bacillus subtilis*, soybean-based fermented foods, complete genome sequence, *Nit1virus*, PGA hydrolase

## Abstract

While the harmful effects of lactic acid bacterial bacteriophages in the dairy industry are well-established, the importance of *Bacillus subtilis*-infecting bacteriophages on soybean fermentation is poorly-studied. In this study, we isolated a *B. subtilis*-infecting bacteriophage BSP10 from *Meju* (a brick of dried fermented soybean) and further characterized it. This *Myoviridae* family bacteriophage exhibited a narrow host range against *B. subtilis* strains (17/52, 32.7%). The genome of bacteriophage BSP10 is 153,767 bp long with 236 open reading frames and 5 tRNAs. Comparative genomics (using dot plot, progressiveMauve alignment, heat-plot, and BLASTN) and phylogenetic analysis strongly suggest its incorporation as a new species in the *Nit1virus* genus. Furthermore, bacteriophage BSP10 was efficient in the growth inhibition of *B. subtilis* ATCC 15245 in liquid culture and in *Cheonggukjang* (a soybean fermented food) fermentation. Artificial contamination of as low as 10^2^ PFU/g of bacteriophage BSP10 during *Cheonggukjang* fermentation significantly reduced bacterial numbers by up to 112 fold in comparison to the control (no bacteriophage). Moreover, for the first time, we experimentally proved that *B. subtilis*-infecting bacteriophage greatly enhanced poly-γ-glutamic acid degradation during soybean fermentation, which is likely to negatively affect the functionalities of *Cheonggukjang*.

## 1. Introduction

*Bacillus subtilis* is a Gram-positive, endospore-forming bacterium commonly found in soil and the human gut [1]. It is an industrially important microorganism used for its probiotic activities in humans [2] and for commercial enzyme production [3]. In addition, *B. subtilis* is one of the major fermenting bacteria in soybean-based fermented foods such as Korean *Cheonggukjang* (a fast-fermented soybean product), *Deonjang* (fermented soybean paste), *Gochujang* (hot red pepper paste), and Japanese *Natto* [4,5]. These types of soybean-based fermented foods are an integral part of the diet of Asian peoples and the market values of these products are increasing day by day [4].

A bacteriophage is a virus that infects host bacteria. Due to recent antibiotic resistance problems, much attention has been paid to the control of harmful bacteria using a bacteriophage [6]. However, the bacteriophage infecting beneficial bacteria are as important as those killing pathogenic bacteria. It is well established that for lactic acid bacteria bacteriophage infection could delay or abort the dairy fermentation process and affect the quality, flavor, and texture of the final products [7,8]. In order to handle the bacteriophage contamination issue in lactic acid bacterial fermentation, a large number of bacteriophage were isolated and characterized, which led to the development of classification schemes and antiphage strategies (adapted factory design, use of phage-resistant starters, and air control) currently in operation [9,10,11]. On the other hand, some studies have been conducted in *B. subtilis*-infecting bacteriophage mostly in Japanese *Natto* [5,12,13,14], and their effects on the fermentation process are not well known.

In this study, we isolated *B. subtilis*-infecting bacteriophage BSP10 from *Meju*, a dried soybean-fermented brick used for the preparation of soybean-based fermented foods (*Gochujang* and *Deonjang*) in Korea. We proved that, during *Cheonggukjang* fermentation, bacteriophage BSP10 could inhibit *B. subtilis* growth and was responsible for the degradation of the functional compound poly-γ-glutamic acid (γ-PGA). Furthermore, we present the complete genome sequence of bacteriophage BSP10 and propose its incorporation as a new species in the genus *Nit1virus*.

## 2. Materials and Methods

### 2.1. Bacterial Strains and Culture Condition

All of the bacteria were grown in Tryptic soy broth (TSB; BD, Sparks, MD, USA) or TSB agar (TSA) plates (1.5% agar) at 37 °C, unless otherwise indicated. Seventy *Bacillus* spp. isolates were used including 52 *B. subtilis* (ATCC [American Type Culture Collection] 19659, ATCC 31028, ATCC 21697, ATCC 15245, ATCC 21556, ATCC 35854, ATCC 21332, ATCC 15841, ATCC 6051, ATCC 14593, ATCC 33677, ATCC 21336, ATCC 21770, ATCC 29233, ATCC 21228, ATCC 23059, ATCC 6633, KCCM [Korean Culture Center of Microorganisms] 11496, KCCM 11731, KCCM 11733, KCCM 11736, KCCM 11738, KCCM 12052, KCCM 12053, KCCM 12150, KCCM 12151, KCCM 12511, KCCM 12512, KCCM 12513, KCCM 40084, KCCM 40820, KCCM 40821, KCCM 41462, KCCM 40088, KCCM 41990, KCCM 41991, KCCM 41992, KCCM 12247, KCCM 11780, KCCM 12248, KCCM 11314, KCCM 11732, KCTC [Korean Collection for Type Cultures] 2217, KACC [Korean Agricultural Culture Collection] 10114, KACC 10112, KACC 17802, KACC 17796, KACC 17797, SRCM 101274, SRCM 100170, SRCM 100336, and SRCM 100169), and 18 other *Bacillus* spp. isolates (9 *B. licheniformis* [JCM 2505, SCC 123050, SCC 122029, SCC 125037, SCD 125015, SCD 122022, SCD 126065, SCD 121044, SRCM 100164], one *B. sphaericus* [JCM 2502], one *B. pumilus* [JCM 2508], one *B. megaterium* [JCM 2506], one *B. weihenstephanensis* [KCTC 3975], one *B. mycoides* [ATCC 21929], one *B. thuringiensis* [ATCC 35866], and three *B. cereus* [ATCC 14579, ATCC 27348, JCM 2152]).

In addition, four non-*Bacillus* bacteria were also used including *Staphylococcus aureus* ATCC 12600, *Staphylococcus xylosus* ATCC 29971, *Escherichia coli* BW25113, and *Enterococcus cloacae* ATCC 13047.

### 2.2. Isolation of B. subtilis-Infecting Bacteriophage BSP10 from Meju

*Meju* was collected from the local market in Korea. The bacteriophage was isolated as described previously [15]. Briefly, 10 g of sample was mixed with 20 mL of TSB and 1 mL of overnight-grown *B. subtilis* ATCC 15245 and incubated at 37 °C with agitation (160 rpm) for 12 h. After the enrichment of *B. subtilis*-infecting bacteriophage, the sample was centrifuged at 12,300× *g* for 10 min and the supernatant was used for bacteriophage isolation. One hundred microliters of the decimally diluted samples in SM buffer (50 mM Tris–HCl, 100 mM NaCl, 10 mM MgSO_4_, pH 7.5) was mixed with overnight-grown *B. subtilis* ATCC 15245 (300 µL) in 4 mL of TA soft agar (0.4% agar in TA broth; 8 g nutrient broth, 5 g NaCl [86 mM], 0.2 g MgSO_4_·7H_2_O [0.8 mM], 0.05 g MnSO_4_ [0.3 mM], and 0.15 g CaCl_2_ [1.0 mM] per 1 L, pH 5.9–6.0) and poured into a pre-solidified TSA plate (1.5% agar). The plates were incubated at 37 °C for 12 h. After incubation, a single clear plaque was picked, eluted with SM buffer and filter-sterilized using a 0.2 µm pore size (ADVANTEC, Tokyo, Japan). The single plaque isolation was repeated twice, and designated as bacteriophage BSP10.

### 2.3. High-Titer Bacteriophage Preparation

Plate elution, large-scale preparation in liquid culture, PEG (polyethylene glycol) precipitation, and CsCl gradient ultracentrifugation of bacteriophage BSP10 were performed as described previously [6], and resulted in a high titer bacteriophage stock preparation (10^11−12^ PFU/mL).

### 2.4. Host Range Analysis by Dotting and Plating Method

Host range analysis was performed initially by dotting 5 µL of PEG-precipitated bacteriophage sample (diluted in SM buffer at a final concentration of 10^7^ PFU/mL) on the TA soft agar containing indicator bacteria. Host infectivity of bacteriophage BSP10 was further confirmed by a plaque forming assay. Briefly, 100 µL of bacteriophage BSP10 (decimally diluted in SM buffer) was mixed with TA soft agar containing 300 µL of the bacteria (dotting positive) and poured into pre-solidified TSA plates followed by incubation at 37 °C. After 24 h incubation, the plaque formations were monitored. The EOP (efficiency of plating, %) was calculated using the following formula, ([average PFU on target bacteria/average PFU on host bacteria *B. subtilis* ATCC 15245] × 100). EOP was classified into three categories, high (>50%), medium (10–50%), and low (<10%).

### 2.5. Transmission Electron Microscopy

Dialyzed CsCl-purified bacteriophage stock was used for transmission electron microscopy, as described earlier with some modifications [15]. Briefly, 10 µL of dialyzed CsCl-purified bacteriophage BSP10 was spotted on the carbon-coated copper grid and incubated for 5 min. The excess solution was removed with filter paper, and the grids were negatively stained with 10 μL of 2% uranyl acetate (Sigma, St. Louis, MO, USA). The samples were visualized by Bio-TEM (Hitachi, Tokyo, Japan) with an acceleration voltage of 100 kV and 200,000× magnification.

### 2.6. One Step Growth Assay

A one-step growth assay was performed as previously described with some modifications [16]. To summarize, the culture of *B. subtilis* ATCC 15245 was grown overnight, added to 25 mL of fresh TA media (1% inoculum) and incubated at 37 °C. When the optical density at 600 nm (OD_600_) was reached at 0.3 (approximately 1 × 10^7^ CFU/mL), the bacteriophage was added at MOI of 0.01 and incubated for 5 min, followed by centrifugation at 26,200× *g* for 10 min. The pellet was resuspended in the same volume of fresh TA medium. Then 1 mL of this mixture was aliquoted into micro-centrifuge tubes (1.5 mL) followed by incubation at 37 °C with shaking (160 rpm). At every 10 min, one tube was taken out from the incubator and centrifuged at 12,300× *g* for 3 min. The supernatant was then collected and used for PFU counting by using TA soft agar.

### 2.7. Genomic DNA Isolation and Sequencing

Genomic DNA was extracted using PEG precipitated bacteriophage stock (filter-sterilized) and an ExgeneTM Cell SV DNA purification kit (GeneAll Biotechnology Co., LTD., Songpa-gu, Korea) after DNase and SDS treatment. Whole genome sequencing was done by Macrogen Inc., Geumcheon-gu, Korea using a MiSeq sequencing system (Illumina, San Diego, CA, USA). Raw reads were trimmed and de novo assembled using A5-miseq pipeline with approximately 575× depth of coverage among the 297,028 reads.

### 2.8. Functional Annotation

Open reading frames (ORF) were predicted using RAST 2.0 [17], Glimmer 3.02 [18], and ORF finder [19]. ORFs were compared with the non-redundant databases using the BLASTP algorithm (default parameter) [20] and functionally annotated. HHpred [21] was used to predict the homing endonucleases in the bacteriophage BSP10 genome. tRNA encoding genes were identified using ARAGORN v1.2.36 [22]. The circular map of bacteriophage BSP10 was prepared using CGView [23]. The whole genome sequence of *Bacillus* bacteriophage BSP10 was deposited in GenBank (Accession number MF422185).

### 2.9. Comparative Genomics and Phylogenetic Positioning of Isolated Bacteriophage

Geneious software v8.14 [24] was used to assess the repeated sequence in the assembled genome of bacteriophage BSP10. DNA identity (%) of bacteriophage BSP10 with other ICTV (International Committee on Taxonomy of Viruses)-classified, large genome-containing (>130 kb), *Bacillus*-infecting bacteriophages [phiNIT1 (NCBI Accession no. AP013029), Grass (KF669652), SPG24 (AB930182), phiAGATE (JX238501), B4 (JN790865), Bastille (JF966203), vB_BceM_Bc431v3 (JX094431), CP-51 (KF554508), W.Ph. (HM144387), and G (JN638751)] was calculated using the BLASTN algorithm and the following equation: ([query coverage × identity]/100) [20,25]. Dot plots of whole genome sequences were generated using Gepard [26]. ProgressiveMauve alignment [27] and CoreGenes (75% threshold) [28] were used to compare the bacteriophage BSP10 genome with the above-mentioned bacteriophage at the DNA and protein level, respectively.

The whole genome sequence of bacteriophage BSP10 was analyzed using the CLuster Analysis of Sequences (CLANS) software package [29] and Gegenees 2.0.0 (tBLASTx method, fragment size −50, step size −25) [30]. The dendrogram was generated by SplitsTree 4.13.1 [31], using the neighbor-joining method based on the similarity matrix generated by Gegenees. In addition, the phylogenetic position of bacteriophage BSP10 was determined by using a major capsid protein, a terminase large subunit, and metallophosphatases [32].

### 2.10. Inhibition of Bacterial Growth in Liquid Culture

Liquid culture inhibition of bacteriophage BSP10 was performed as described by Shin, Bandara, Shin, Ryu, and Kim [6], with some modifications. Briefly, 1% of overnight grown *B. subtilis* ATCC 15245 was added to 25 mL fresh TSB media and incubated at 37 °C with shaking (160 rpm). Bacteriophage BSP10 was added at a multiplicity of infection (MOI) of 1 and 0.1 when OD_600_ was approximately 0.3. Bacterial growth (either colony forming units [CFU] counts or OD_600_ using spectrophotometer [Biochrom Libra S22 Visible Spectrophotometer, Cambourne, Cambridge, UK]) and plaque forming units (PFU) were monitored at the designated time points for 72 h. The experiment was performed for three times and the values were represented as the mean ± Standard Deviation (SD).

### 2.11. Inhibition of Bacterial Growth in Cheonggukjang

*B. subtilis* ATCC 15245 growth inhibition by bacteriophage BSP10 was evaluated by modifying the method used for *B. cereus* inhibition by bacteriophage in *Cheonggukjang* [6]. Washed soybeans were immersed in distilled water for 10 h at 4 °C. After removing the water, the beans were air-dried at room temperature for 30 min. Ten grams of beans was put into a 100 mL conical flask and autoclaved at 121 °C for 15 min. After cooling the flask at room temperature, *B. subtilis* ATCC 15245 was added at a concentration of 10^4^ CFU/g (which is the standard concentration used in factories) in 1 mL of TA, followed by artificial contamination of bacteriophage BSP10 at 10^2^, 10^4^, and 10^6^ PFU/g. The flasks were incubated at 37 °C for 72 h without any perturbation. *B. subtilis* growth was monitored using TSA and the bacteriophage count was checked using TA soft agar, as stated above, every 24 h. Data were statistically analyzed by using one-way ANOVA (Analysis of variance) and Duncan’s multiple range test in Sigma Plot 12.5 (Systat Software, San Jose, CA, USA) to determine the significant relationship between the mean values. The experiment was carried out three times and the values were represented as the mean ± SD.

### 2.12. Poly-Gamma-Glutamic Acid (γ-PGA) Analysis in Cheonggukjang

γ-PGA was extracted by modifying the method described by Zeng, et al. [33]. Briefly, 10 g of *Cheonggukjang* (24 h incubation sample) was mixed with 20 mL of deionized water followed by shaking at 160 RPM for 1 h at 4 °C. After removing the cells and solid particles by centrifugation at 16,350× *g* for 20 min, the supernatant was mixed with 4 volumes of cold ethanol. The sample was then centrifuged at 16,350× *g* for 20 min to precipitate γ-PGA. The ethanol was allowed to evaporate and the dried precipitate was dissolved in 1 mL of deionized water, followed by the addition of 2 µL of DNase (5 U/µL) and incubation at 37 °C for 1 h to degrade the bacterial DNA.

Extracted γ-PGA and a standard poly-L-γ-glutamic acid sodium salt (molecular weight ≥750 kd; Sigma, USA) were electrophoresed following the method of Mamberti, et al. [34]. Briefly, the samples were mixed with 1 µL loading dye (5 mg/mL Bromophenol Blue, 50% [*v*/*v*] Glycerol in TAE buffer) and allowed to electrophorese in 1.5% agarose gel in TAE buffer (40 mM Tris [pH 7.6], 20 mM Acetic acid, and 1 mM EDTA) at 50 V for 90 min. γ-PGA was visualized by staining the gel with 0.5% methylene blue in 3% acetic acid for 30 min followed by destaining in H_2_O.

### 2.13. Determination of Poly-γ-Glutamate Hydrolase (γ-PGA Hydrolase) Activity

In order to find out the source of the γ-PGA hydrolase enzyme, the bacterial culture supernatant (without bacteriophage infection), cytosolic contents of the bacteria (without bacteriophage infection), and bacteriophage-infected bacterial lysate were used for γ-PGA hydrolase enzyme assay. For bacterial culture supernatant preparation, *B. subtilis* ATCC 15245 were grown in TSB media at 37 °C in shaking condition (160 rpm) for 24 h followed by centrifugation at 26,200× *g* for 10 min. The cytosolic contents of the bacteria were prepared after lysis of the control bacteria (without bacteriophage infection) by lysozyme (at 37 °C for 1 h) and sonication (Vibra-Cell™ Ultrasonic Liquid Processors, Newtown, CT, USA) for 10 min, followed by centrifugation at 12,300× *g* for 5 min to remove the cell debris. The bacteriophage-infected bacterial lysate was prepared by the infection of bacteriophage BSP10 (MOI 1) to *B. subtilis* ATCC 15245 culture in TSB media followed by incubation at 37 °C for 24 h in shaking condition (160 RPM), and the supernatant was collected after centrifugation at 26,200× *g* for 10 min.

γ-PGA hydrolase activity was assayed following the method of Kimura and Itoh [13]. Briefly, 500 µL of the reaction mixture (1 mg of γ-PGA, 10 mM sodium phosphate [pH 7.5], 150 mM NaCl, and 200 µL supernatant) was incubated at 37 °C for 1 h. Then, 2 µL of DNase (5 U/µL) was added to each tube and incubated at 37 °C for 30 min to degrade the bacterial DNA. The samples were resolved in 1.5% agarose gel as stated above.

## 3. Results

### 3.1. Isolation, Morphology, Host Range Analysis, and One Step Growth Curve of Bacteriophage BSP10

**Figure 1:**
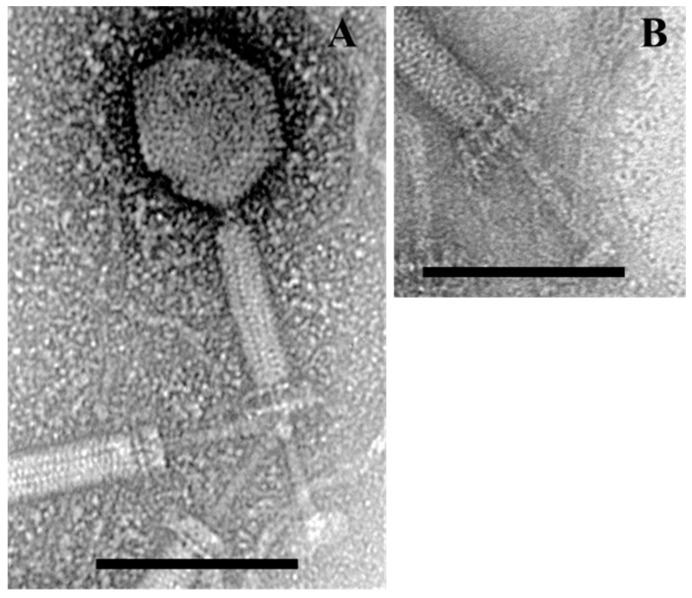
Transmission electron micrographs of *Bacillus* bacteriophage BSP10. Contracted state of bacteriophage BSP10 (**A**); baseplate structure upon tail contraction (**B**). The scale bar corresponds to 100 nm.

Bacteriophage BSP10 was isolated from a fermented soybean brick, *Meju*, by using *B. subtilis* ATCC 15245 as a host. The bacteriophage showed a clear plaque in TA soft agar after 12 h of incubation at 37 °C. Bacteriophage BSP10 was found to be a member of the *Myoviridae* family with an isometric head (82 ± 8 nm long in diameter; *n* = 6) and a contractile tail (length, 180 ± 10 nm; width, 24 ± 1 nm, *n* = 6) (Figure 1). Similar to the other SPO1-related phages, two parallel interconnected planes perpendicular to the tail were clearly observed upon contraction (Figure 1A,B).

Bacteriophage BSP10 showed a narrow host range against *B. subtilis* (17 out of 52 strains) (Table S1). Depending on the plaque formation ability of bacteriophage BSP10 in different hosts (*B. subtilis*), EOP was classified as high (>50%, 9 strains), medium (10–50%, 4 strains), and low (<10%, 4 strains). Other tested bacterial strains (closely-related *B. licheniformis*, other *Bacillus*, and non-*Bacillus* Gram positive/negative bacterial species) were not infected by bacteriophage BSP10.

**Figure 2:**
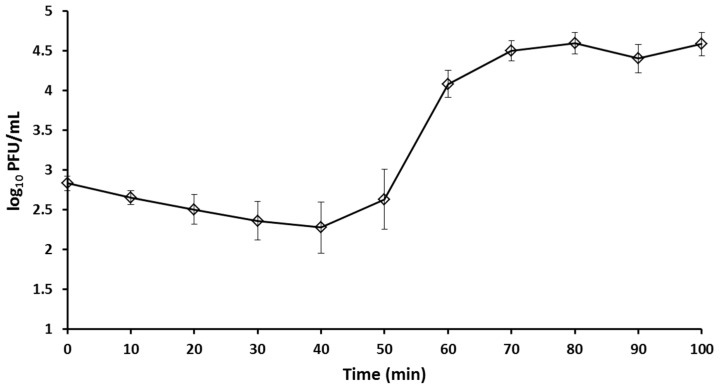
One step growth curve of bacteriophage BSP10. Error bars represent standard deviation.

The one-step growth curve of bacteriophage BSP10 showed that the latent period was 40 min and burst size was approximately 185 in the propagation strain, *B. subtilis* ATCC 15245 (Figure 2).

### 3.2. Complete Genome Sequencing and Functional Annotation of Bacteriophage BSP10

**Figure 3:**
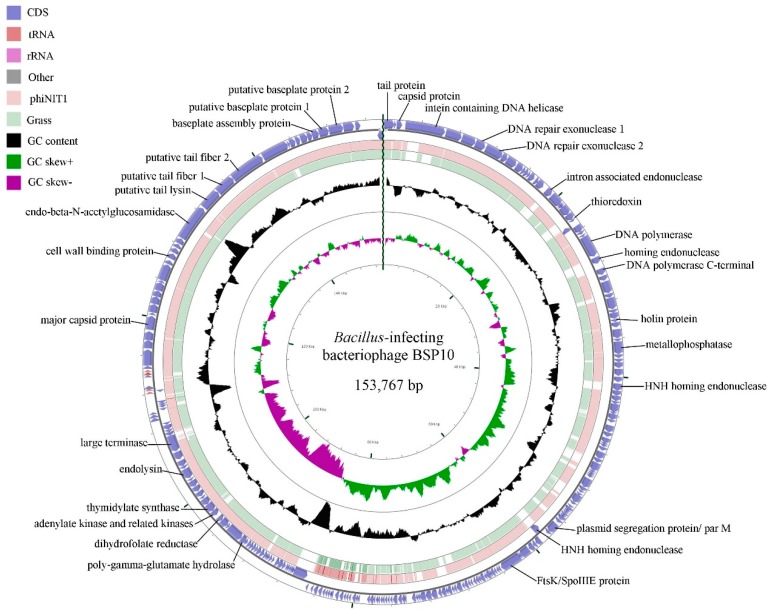
BLASTN genome comparison of bacteriophage BSP10 and the other members (phiNIT1 and Grass) of the genus, *Nit1virus*. The outer ring represents the ORFs of the bacteriophage BSP10 genome (blue). BLASTN homologies between bacteriophage BSP10 with phiNIT1 (light pink) and Grass (light green) were shown in the other two rings. Some major putative gene functions are labeled.

The genome of bacteriophage BSP10 is 153,767 bp long with 42.1% G+C content. A higher read coverage (~2 fold) was observed in the region between nucleotides 78,776–84,660 (5885 bp) using Geneious software, suggesting the presence of a long terminal repeat similar to phiNIT1 and Grass in the *Nit1virus* genus [25,35,36]. A total of 236 ORFs (185 on the forward strand and 51 on reverse strand) and 5 tRNAs were predicted (Figure 3). Among them, 57 ORFs (24.2%) were functionally annotated using the BLASTP algorithm and they were categorized into 7 groups (Table S2), including structural proteins, DNA replication, transcription and repair, nucleotide metabolism, DNA packaging, introns and homing endonuclease, host lysis, and other functions. No genes for integrase and antibiotic resistance were identified.

### 3.3. Comparative Genomics and Phylogenetic Position

The bacteriophage BSP10 genome showed 87.30%, 84.48%, and 92.12% nucleotide sequence homology (BLASTN) with the ICTV-classified members of *Nit1virus* genus, phiNIT1, Grass, and SPG24, respectively, whereas no significant similarity was observed with the representative members of the genus *Agatevirus*, *B4virus*, *Bastillevirus*, *Bc431virus*, *Cp51virus*, or *Wphvirus* (Table 1). It also exhibited 75.42% and 83.9% proteomic homology with the bacteriophage phiNIT1 and Grass, respectively (the proteomic homology of bacteriophage BSP10 and SPG24 was not calculated as the genome of bacteriophage SPG24 was found under-annotated in the NCBI database), and less than 50% homology with the other bacteriophages (Table 1) in the CoreGenes analysis.

**Figure 4:**
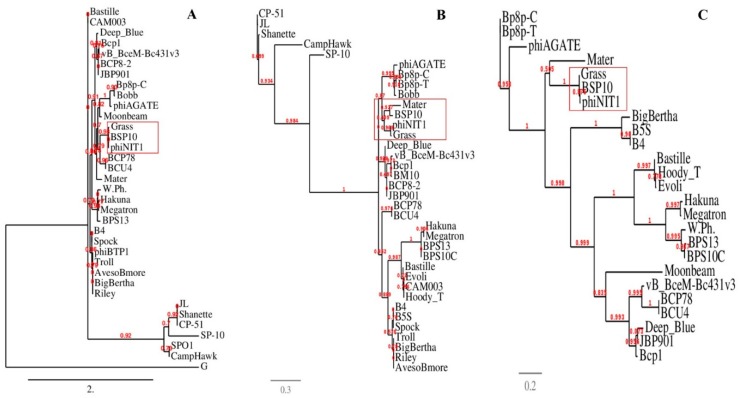
Phylogenetic analysis of the major capsid proteins (**A**), terminase large subunit (**B**), and metallophosphatases (**C**) of ICTV-classified *Bacillus* bacteriophages. The tree was constructed using ‘‘one click’’ within the phylogeny.fr program. The approximate likelihood ratios for individual branches are shown in numbers.

When the bacteriophage BSP10 genome was compared with the other ICTV-classified *Bacillus* bacteriophages using CLANS, it was clustered with the members of previously-proposed Bastille-like group bacteriophages [37] (Figure S1). Genome comparison was conducted by using a dot plot matrix and progressiveMauve alignment which showed a similar genomic arrangement between bacteriophage BSP10 and the other members of the *Nit1virus* genus (Figures S2 and S3). In addition, heat-plot analysis of the translated protein comparison of whole bacteriophage genomes exhibited high similarities among the members of the *Nit1virus* genus and bacteriophage BSP10 (Figure S4). Similarly, the phylogenetic tree based on heat-plot (Figure S4) and the selected protein markers such as major capsid protein, terminase large subunit, and metallophosphatases (Figure 4), also grouped bacteriophage BSP10 with the members of the genus *Nit1virus*.

BLASTP comparison of bacteriophage BSP10 ORFs showed that 75 (31.8%) and 48 (20.3%) of ORFs had a high resemblance (≥99%) with bacteriophage phiNIT1 and Grass, respectively. On the other hand, 25 and 32 ORFs of bacteriophage BSP10 were found to be unique (spanned throughout the genome) compared with phiNIT1 and Grass, respectively, while 16 ORFs of phiNIT1 and 33 ORFs of Grass are absent in bacteriophage BSP10 genome (again, as the genome of bacteriophage SPG24 was not annotated properly, the comparison was not made).

### 3.4. Identification of Poly-Gamma-Glutamate Hydrolase Homolog Gene in Bacteriophage BSP10 Genome

The bacteriophage BSP10 genome contained a poly-gamma-glutamate hydrolase (γ-PGA hydrolase) homolog (ORF172, 208 amino acids), which might function to breakdown γ-PGA, which is responsible for the sticky appearance as well as the functional properties of soybean-based fermented foods. It showed high similarities (BLASTP) with those of Grass (NCBI protein id. YP_008771398.1, 99%) and phiNIT1 (NCBI protein id. YP_008318292.1, 98%).

### 3.5. Growth Inhibition of B. subtilis ATCC 15245 and Growth of Bacteriophage BSP10 in Liquid Culture

**Figure 5:**
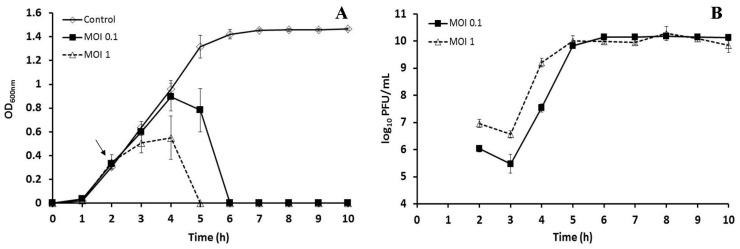
Growth inhibition of *B. subtilis* ATCC 15245 in liquid culture by bacteriophage BSP10 (**A**) and propagation of bacteriophage BSP10 during liquid culture inhibition (**B**). *B. subtilis* ATCC 15245 was grown in TSB liquid media and bacteriophage BSP10 was added at MOIs of 1 and 0.1 after 2 h of bacterial inoculation. The data were presented as mean ± SD.

Bacteriophage BSP10 effectively inhibited the growth of *B. subtilis* ATCC 15245 in liquid culture. Clear lysis was observed 3 h postinfection for MOI 1 (OD_600_ of 0.00) and 4 h postinfection for MOI 0.1 (0.00), whereas the OD_600_ of the control (no bacteriophage addition) was 1.32 ± 0.10 after 5 h (corresponding to 3 h postinfection in the bacteriophage-treated sample) and 1.42 ± 0.04 after 6 h (corresponding to 4 h postinfection) of bacterial inoculation (Figure 5A).

After 24 h (48 and 72 h) incubation, the bacterial CFU counts were 8.6 ± 0.2 (8.0 ± 0.4 and 7.7 ± 0.1 log_10_ CFU/mL) and 3.1 ± 1.3 log_10_ CFU/mL (4.0 ± 0.6 and 3.9 ± 0.6 log_10_ CFU/mL) for the control (without bacteriophage) and the MOI 0.1 treated set, respectively. Interestingly, no bacterial regrowth was detected for MOI 1 after 24, 48, and 72 h. With regard to bacteriophage growth, PFU counts rapidly increased, and after 3 h postinfection reached 10.0 ± 0.2 log_10_ PFU/mL (MOI 1) and 9.8 ± 0.1 log_10_ PFU/mL (MOI 0.1) (Figure 5B). No significant changes in the PFU counts were observed for the next 72 h.

### 3.6. B. subtilis ATCC 15245 Growth Inhibition by Bacteriophage BSP10 in Cheonggukjang Fermentation

**Figure 6:**
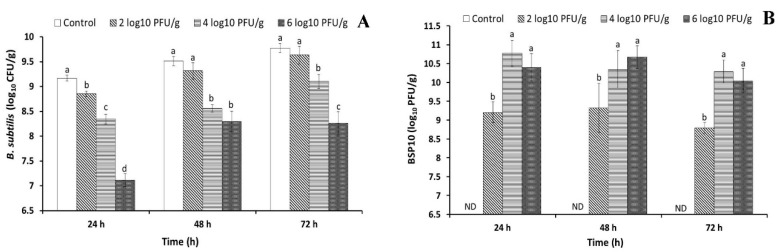
Changes of *B. subtilis* ATCC 15245 counts in the presence of bacteriophage BSP10 (**A**) and propagation of bacteriophage BSP10 during *Cheonggukjang* fermentation (**B**). *Cheonggukjang* was prepared by *B. subtilis* ATCC 15245 (10^4^ CFU/g) and artificial contamination of bacteriophage BSP10 at a concentration of 0 (control), 10^2^, 10^4^, and 10^6^ PFU/g followed by incubation at 37 °C for a period of 72 h. At the end of every 24 h period, *B. subtilis* growth was monitored using TSA and bacteriophage counts was checked using TA soft agar, as described in Materials and Methods section. Data were presented as mean ± SD. Different superscript letters in each segment are significantly different (*p* < 0.05) according to ANOVA (Duncan’s multiple range tests). ND: Not detected.

The fast-fermented soybean product *Cheonggukjang* was prepared to check the effect of bacteriophage BSP10 on *B. subtilis* growth inhibition during soybean fermentation. Bacteriophage BSP10 was artificially contaminated at 10^2^, 10^4^, and 10^6^ PFU/g in autoclaved soybean which contained 10^4^ CFU/g (which is the standard concentration used in factories) of *B. subtilis* ATCC 15245. After 24 h incubation, statistically significant differences (*p* < 0.05) were observed among all the treatments and the bacterial numbers were 8.85 ± 0.06, 8.34 ± 0.10, and 7.11 ± 0.13 log_10_ CFU/g for 10^2^, 10^4^, and 10^6^ PFU/g addition of bacteriophage BSP10, respectively, as compared to 9.16 ± 0.04 log_10_ CFU/g of the control (no bacteriophage) (Figure 6A). *B. subtilis* counts increased in all treatments after 48 h incubation, but the numbers were significantly lower in 10^4^ PFU/g (8.56 ± 0.08 log_10_ CFU/g) and 10^6^ PFU/g (8.29 ± 0.21 log_10_ CFU/g) of bacteriophage BSP10-treated sets as compared to control (9.51 ± 0.09 log_10_ CFU/g) and 10^2^ PFU/g treated sets (9.31 ± 0.16 log_10_ CFU/g) (Figure 6A). Similarly, after 72 h, bacterial numbers increased in all of the sets (except in 10^6^ PFU/g treated flasks). No statistically significant difference in the bacterial numbers was observed between control (9.78 ± 0.09 log_10_ CFU/g) and 10^2^ PFU/g bacteriophage BSP10-treated set (9.63 ± 0.18 log_10_ CFU/g), whereas the numbers were significantly different in 10^4^ PFU/g (9.10 ± 0.14 log_10_ CFU/g) and 10^6^ PFU/g (8.26 ± 0.23 log_10_ CFU/g) treated sets as compared to the control (Figure 6A).

### 3.7. Bacteriophage BSP10 Growth during 72 h Cheonggukjang Fermentation

The bacteriophage numbers increased rapidly during first 24 h incubation and the counts were 9.21 ± 0.30, 10.77 ± 0.34, and 10.40 ± 0.37 log_10_ PFU/g for an initial bacteriophage contamination level of 10^2^, 10^4^, and 10^6^ PFU/g, respectively (Figure 6B). The numbers were not much changed after 48 h incubation reaching 9.33 ± 0.66, 10.34 ± 0.50, and 10.67 ± 0.30 log_10_ PFU/g for 10^2^, 10^4^, and 10^6^ PFU/g, respectively (Figure 6B). After 72 h incubation, the bacteriophage numbers did not change, and were similar to the 48 h incubation numbers of all treatments (8.80 ± 0.14, 10.29 ± 0.30, and 10.04 ± 0.33 log_10_ PFU/g for 10^2^, 10^4^, and 10^6^ PFU/g, respectively) (Figure 6B).

### 3.8. Degradation of γ-PGA in Cheonggukjang and γ-PGA Hydrolase Assay

**Figure 7:**
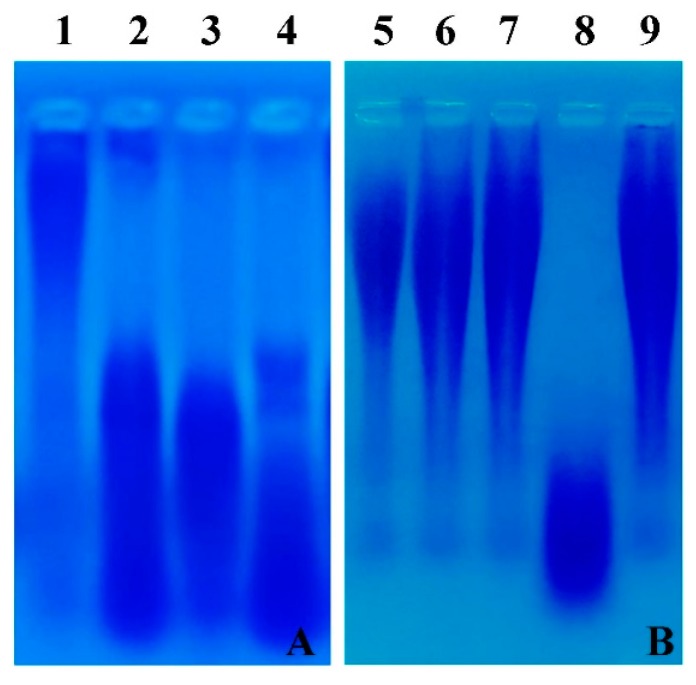
(**A**) Degradation of γ-PGA in *Cheonggukjang* samples. Extracted γ-PGA was electrophoresed in 1.5% agarose gel, followed by staining with 0.5% methylene blue in 3% acetic acid and destaining with distilled water. Lane 1: control (without bacteriophage), lane 2: 10^2^ PFU/g of bacteriophage BSP10, lane 3: 10^4^ PFU/g of bacteriophage BSP10, lane 4: 10^6^ PFU/g of bacteriophage BSP10. (**B**) γ-PGA hydrolase activity in the liquid culture. Standard γ-PGA (MW ≥ 750 kd) was incubated with bacterial culture supernatant (no bacteriophage infection), cytosolic content of the bacteria (no bacteriophage infection), and bacteriophage-infected bacterial lysate, and electrophoresed in 1.5% agarose gel as stated above. Lane 5: standard γ-PGA (MW ≥ 750 kd), lane 6: γ-PGA treated with distilled water, lane 7: γ-PGA treated with bacterial culture supernatant (without bacteriophage infection); lane 8: γ-PGA treated with bacteriophage BSP10-treated culture supernatant; lane 9: γ-PGA treated with the cytosolic content of *B. subtilis* ATCC 15245 (control, without bacteriophage infection).

To check the degradation of γ-PGA in *Cheonggukjang*, γ-PGA was extracted from the bacteriophage-treated and -untreated *Cheonggukjang* samples, and electrophoresed in 1.5% agarose gel. The fragmentation of γ-PGA (low molecular weight) was observed in the bacteriophage-treated *Cheonggukjang* samples, while a clear band of high molecular weight γ-PGA was noticed for the bacteriophage-untreated sample (Figure 7A).

γ-PGA hydrolase activity was undetectable in the bacterial culture supernatant (no bacteriophage infection) and the bacterial cytosolic content. On the other hand, the degraded γ-PGA was observed in the bacteriophage-treated bacterial lysate (Figure 7B).

## 4. Discussion

As previously acknowledged, *B. subtilis* is used in different soybean-based fermented food industries, and is responsible for the fermentation and qualities of the final products [4,5]. Considering the similar roles of lactic acid bacterial bacteriophages in the dairy industry, it can be assumed that contamination by a *B. subtilis*-infecting bacteriophage might create a serious problem, such as the delay of fermentation process leading to economic losses and changes in the functional properties of the final product. The knowledge about the prevalence, diversity, classification, and properties of bacteriophages is essential for the fermentation industry to develop antiphage strategies and minimize the risk of bacteriophage infections [8]. The availability of sufficient genome sequences of lactic acid bacteria-infecting bacteriophages has helped in the establishment of the taxonomy and PCR-based detection tools for the lactic acid bacterial bacteriophage in dairy environments [9,10]. In contrast, relatively few *B. subtilis*-infecting bacteriophage whole genome sequences (24 sequences including bacteriophage BSP10) are available in the NCBI database until now.

In this study, we have isolated a *Myoviridae* family bacteriophage, BSP10 from a dried soybean-fermented brick, *Meju* (Figure 1). Bacteriophage BSP10 exhibited high sequence homologies and similar genomic arrangements (whole genome comparison using dot plot matrix and progressiveMauve alignment) with the members of the *Nit1virus* genus (phiNIT1, Grass, and SPG24) (Figures S2 and S3). Moreover, in phylogenetic analysis (using Phylogeny.fr and SplitsTree programs), bacteriophage BSP10 was clustered with the members of the *Nit1virus* genus (Figure 4 and Figure S4). The above-mentioned findings clearly suggest that bacteriophage BSP10 belongs to the *Nit1virus* genus.

According to the current ICTV classification [25], 95% DNA sequence identity is the criterion for the demarcation of a new species in the genus *Nit1virus*. The whole genome sequence comparison (BLASTN) of bacteriophage BSP10 with phiNIT1 (87.30%), Grass (84.48%), and SPG24 (92.12%) (Table 1), suggests that bacteriophage BSP10 is a new species in the *Nit1virus* genus. Incorporation of this new species will further help to develop a better taxonomy of *B. subtilis*-infecting bacteriophage.

Mobile elements are prevalent in *B. subtilis*-infecting bacteriophages (SPO1 [38], such as CampHawk [39] and vB_BsuM-Goe2 [Accession no. KY368639.1]), including the members of the *Nit1virus* genus. Bacteriophage BSP10 encodes four homing endonucleases (ORF18, GIY-YIG family; ORF30, 48 and 85, HNH family) (Table S3) and one intein sequence (disrupting DNA helicase I, ORF4). On the other hand, bacteriophages phiNIT1 and Grass each contain only one HNH homing endonuclease (ORF183a [42% BLASTP identity with ORF85 of bacteriophage BSP10] and ORF132 [40% BLASTP identities with ORF30 of bacteriophage BSP10], respectively), while no intein sequence is identified. Considering the high similarities in the genomes of the same genus, the number and distribution of mobile elements, including homing endonuclease and intein sequences, may represent the uniqueness of each phage among the members of the *Nit1virus* genus.

Previously, it was proposed that the genes within the terminal redundancy region of the bacteriophage are the first to be expressed in the infected cell [40]. It was also suggested that their nucleotide sequence and the transcriptional units (e.g., promoters) might be optimized for efficient early expression in the host environment [40,41]. In this study, we found that the high read portion (suggesting the presence of long terminal repeat [35]) in the genome of bacteriophage BSP10 was relatively more diverse (84.39%, 71.04%, and 82.32% in BLASTN with the bacteriophages phiNIT1, Grass, and SPG24, respectively) than the other parts of the genomes (87.3%, 85.44%, and 92.12% with phiNIT1, Grass, and SPG24, respectively). Moreover, in this region, the bacteriophage BSP10 genome contains 4 unique ORFs which are absent in the genomes of bacteriophage phiNIT1 and Grass (annotation of SPG24 is incomplete). While a detailed study is needed to find out the role of the individual genes, it can be hypothesized that the genes in the redundancy region (bacteriophage BSP10 genome) might be naturally selected or evolved for efficient early expression in the host environment which endows diversity and even uniqueness to the bacteriophage.

*Nit1virus* bacteriophages were found in a wide range of environments (phiNIT1 and BSP10, soybean-based fermented foods; Grass, soil samples; SPG24, decayed rice hay manure as mentioned in the Genbank file) and different geographical locations (phiNIT1, Japan; Grass, USA; SPG24 and BSP10, Korea) [13,36]. Considering the survivability in different environmental conditions of *Nit1virus* bacteriophages, it can be postulated that these widespread bacteriophages might be a threat to soybean-based fermented food industries and other industries where *B. subtilis* is used as a fermenting bacteria.

Along with the genome sequence analysis, morphological features are also important to classify the bacteriophage. The overall morphological features of bacteriophage BSP10 were similar to that of phiNIT1 (TEM images of other members in the *Nit1virus* genus are not available), possessing a long, contractile tail and an isometric head; baseplates moved upwards and the tail tube was extended from the baseplates and tail sheath (Figure 1). On the other hand, the dimensions of bacteriophage BSP10 (Figure 1) were found to be smaller (head diameter, 82 nm; and tail length, 180 nm) than phiNIT1 (head diameter, 100 nm; and tail length, 250 nm) [42]. Morphological variations between species of the same genus were also observed in the *Bc431virus* genus; the tail length of the species *Bacillus virus JBP901* was 170 ± 5 nm [6], whereas that of another species *Bacillus virus BCP82* was 210 nm on average [15]. The importance of this size difference among the different species in the *Nit1virus* genus is currently not clear.

The latency period (40 min) and burst size of bacteriophage BSP10 (approximately 185) (Figure 2) were found to be significantly different in comparison with the *B. subtilis*-infecting *Myoviridae* family bacteriophage, phiNIT1 (latency period, 30 min; burst size approximately 50) [42], SPO1 (latency period, 80 min; burst size 70) [43], and vB_BsuM-Goe3 (latency period, 55 min; burst size approximately 114) [44]. The distinct experimental conditions might be the reasons for the large variation in the latency period and burst sizes as suggested by the other researchers [15,43,44].

Many studies explored the beneficial uses of bacteriophage to control pathogenic bacteria such as *Listeria monocytogenes*, *Escherichia coli*, *Salmonella* spp., *Campylobacter* spp., *Enterobacter sakazakii*, and *B. cereus* [15,45,46,47]. In contrast, the detrimental effects of the bacteriophage (lactic acid bacterial bacteriophages could delay or abort dairy fermentation by constant lysis of fermentative bacteria) were also reported [7,8]. We observed that bacteriophage BSP10 efficiently inhibited the growth of *B. subtilis* ATCC 15245 in liquid culture (Figure 5A) as well as in *Cheonggukjang* (Figure 6A). Significant reductions in bacterial numbers were observed (up to 112 folds) for 10^4^ and 10^6^ PFU/g treated sets throughout the *Cheonggukjang* fermentation (Figure 6A). Even 10^2^ PFU/g of bacteriophage BSP10 decreased bacterial numbers (2 fold) after 24 h (Figure 6A). Hence, it is clear that a very low number of bacteriophage can hamper bacterial growth and could delay or abort the soybean fermentation process.

In spite of the presence of high numbers of bacteriophage after 24 h, the bacterial counts gradually increased in all the sets at 48 and 72 h incubation, and no significant differences in bacterial numbers were observed for 10^2^ PFU/g of bacteriophage BSP10-treated sets and the control at 48 and 72 h (Figure 6A). It can be articulated that the incomplete inhibition of *B. subtilis* might be due to either the solid-state growth media where all of the bacteria may not be accessed by bacteriophage or the development of bacterial resistance. Foschino et al. [48] also stated a more effective growth inhibition of *Lactobacillus sanfranciscensis* by bacteriophage in liquid sourdough fermentation than the solid-state fermentation.

High bacteriophage titer was observed during the liquid culture inhibition (Figure 5B) and *Cheonggukjang* fermentation (Figure 6B), possibly due to the large burst size of bacteriophage BSP10 (~185, Figure 2) and/or constant presence of an adequate number of the host in the medium [49,50]. Although no reports have described the growth of *B. subtilis*-infecting bacteriophage during solid-state soybean fermentation, our findings might be comparable to the high titers of lactic acid bacterial bacteriophage in whey samples (10^9^–10^10^ PFU/mL) collected after cheese fermentation [51].

γ-PGA, a sticky material, is produced in soybean-based fermented foods by bacterial (especially *B. subtilis*) fermentation [52]. It has several health-beneficial properties such as total cholesterol reduction, immune function enhancement, increased calcium absorption, and antitumor effects [53,54,55]. On the other hand, it was assumed that it might protect the bacteria from bacteriophage infection [13]. A number of bacteriophage genomes were reported to encode a putative γ-PGA-degrading enzyme, and recently, a purified γ-PGA hydrolase from phiNIT1 was shown to be able to actually breakdown γ-PGA [34,42]. However, to the best of our knowledge, whether or not *B. subtilis*-infecting bacteriophage directly affects γ-PGA qualities during fermentation in the soybean medium has not been experimentally proven.

In this study, we found the fragmentation of γ-PGA in the bacteriophage BSP10-treated samples during *Cheonggukjang* fermentation (Figure 7A). Furthermore, we proved that γ-PGA hydrolase activity was not detected in the bacterial culture supernatant (no bacteriophage infection) or bacterial cytosolic contents (no bacteriophage infection) but in the bacteriophage-infected bacterial lysate (Figure 7B). Taken together, our data strongly suggest that γ-PGA hydrolase activity is responsible for the degradation of γ-PGA and the enzyme (likely to be encoded by ORF172) is originated from the bacteriophage BSP10.

The production of a low molecular weight γ-PGA in bacteriophage-treated samples could negatively affect the functionalities of γ-PGA. Taken together with the reports on the detrimental effects of lactic acid bacterial bacteriophage in dairy environments, such as reduced acidification of milk in presence of lactic acid bacterial bacteriophage [7,56], our findings suggest that the contamination of *B. subtilis*-infecting bacteriophage in *Cheonggukjang* fermentation not only retards bacterial growth but also significantly lowers product qualities.

## 5. Conclusions

A *Myoviridae* family *B. subtilis*-infecting bacteriophage BSP10 was isolated from *Meju* and found to be a new species in the *Nit1virus* genus. We proved that bacteriophage BSP10 infection in *Cheonggukjang* fermentation not only affected bacterial growth but also decreased the quality of the soybean-based fermented food by degrading the functional compound, γ-PGA. This study will provide useful information for future research on the host–bacteriophage interaction and help to establish a clear classification scheme of *B. subtilis*-infecting bacteriophage.

## Figures and Tables

**Table 1:** Genomic and proteomic features of ICTV-classified *Bacillus* bacteriophages and bacteriophage BSP10.

Genus	Bacteriophage	GenBank Accession No.	Genome Length (bp)	Genome (G+C mol%)	No. of CDS	No. of tRNAs	DNA Sequence Identity % ^a^	Percent Homologous Proteins ^b^
	BSP10	MF422185	153,767	42.1	236	5	100	100
*Nit1virus*	phiNIT1	AP013029	155,631	42.1	219	4	87.30	75.42
	Grass	KF669652	156,648	42.2	252	3	84.48	83.9
	SPG24 ^c^	AB930182	152,069	42.2	34	4	92.12	-
*Agatevirus*	phiAGATE	JX238501	149,844	41.0	210	4	0.74	46.19
*B4virus*	B4	JN790865	162,596	37.7	277	0	0	39.83
*Bastillevirus*	Bastille	JF966203	153,962	38.1	273	7	0	37.71
*Bc431virus*	vB_BceM_Bc431v3	JX094431	158,621	40.0	238	21	0.72	42.8
*Cp51virus*	CP-51	KF554508	138,658	40.9	221	2	0	16.53
*Wphvirus*	W.Ph.	HM144387	156,897	36.4	274	3	0	33.47
Unassigned	G	JN638751	497,513	29.9	675	18	0	15.25

^a^ DNA sequence identity was calculated by using the BLASTN algorithm and the following equation: [(query coverage × identity)/100]; ^b^ Percent homologous proteins were calculated using CoreGenes; ^c^ Not properly annotated till date.

## Supplementary Materials

The following are available online at http://www.mdpi.com/1999-4915/10/5/240/s1, Figure S1: CLANS analysis of a total of 10 ICTV (International Committee on Taxonomy of Viruses)-classified *Bacillus* bacteriophages (phiNIT1, Grass, SPG24, phiAGATE, B4, Bastille, vB_BceM_Bc431v3, CP-51, W.Ph., and G), and bacteriophage BSP10. Edge weights were calculated from the P values of the BLASTN high-scoring segment pairs (e-value cut-off = 1e^−5^). The network was visualized after 25,000 runs using the CLANS software package; Figure S2: Whole-genome nucleotide dot plot of bacteriophage BSP10 and other ICTV (International Committee on Taxonomy of Viruses)-classified *Bacillus* bacteriophage (phiNIT1, Grass, SPG24, phiAGATE, B4, Bastille, vB_BceM_Bc431v3, CP-51, W.Ph., and G). Dot plots of whole genome sequences were generated using Gepard program; Figure S3: Bacteriophage BSP10 genome is compared with genus *Nit1virus* (bacteriophage phiNIT1, Grass, and SPG24), *Agatevirus* (phiAGATE), *B4virus* (B4), *Bastillevirus* (Bastille), *Bc431virus* (vB_BceM_Bc431v3), *Cp51virus* (CP-51), and *Wphvirus* (W.Ph.) as well as unassigned phage G using progressiveMauve alignment. Colored blocks indicate 1 to 1 best alignment and the sequence similarity is indicated by similarity plot within colored blocks; Figure S4: Heat-plot showing the results of genome comparison of bacteriophage BSP10 with other ICTV-classified *Bacillus* phage and the resulting phylogenetic tree. The similarity values were calculated using Gegenees software based on pairwise translated comparison of the analyzed sequences (tBLASTx method, fragment size −50, step size −25, threshold 10%). The heat plot colors reflect this similarity, ranging from low (red) to high (green). The tree was constructed with SplitsTree using the neighbor-joining method. The scale bar represents a 10% difference in the average tBLASTx score; Table S1: Sensitivity of *Bacillus subtilis* strains against *Bacillus* bacteriophage BSP10; Table S2: Functional grouping of annotated ORFs in *Bacillus* bacteriophage BSP10; Table S3: Homing endonuclease in bacteriophage BSP10 genome predicted by HHpred program.
