# Supplementary material for: Complete Nucleotide Sequence Analysis of a Novel Bacillus subtilis-Infecting Bacteriophage BSP10 and Its Effect on Poly-Gamma-Glutamic Acid Degradation
Source: Viruses. 2018 May 4;10(5):240. doi: 10.3390/v10050240 (PMC5977233; doi:10.3390/v10050240)
Supplement: Supplementary file 1 [file viruses-10-00240-s001.zip › Supplementary Figures.pdf]

Supplementary Figures

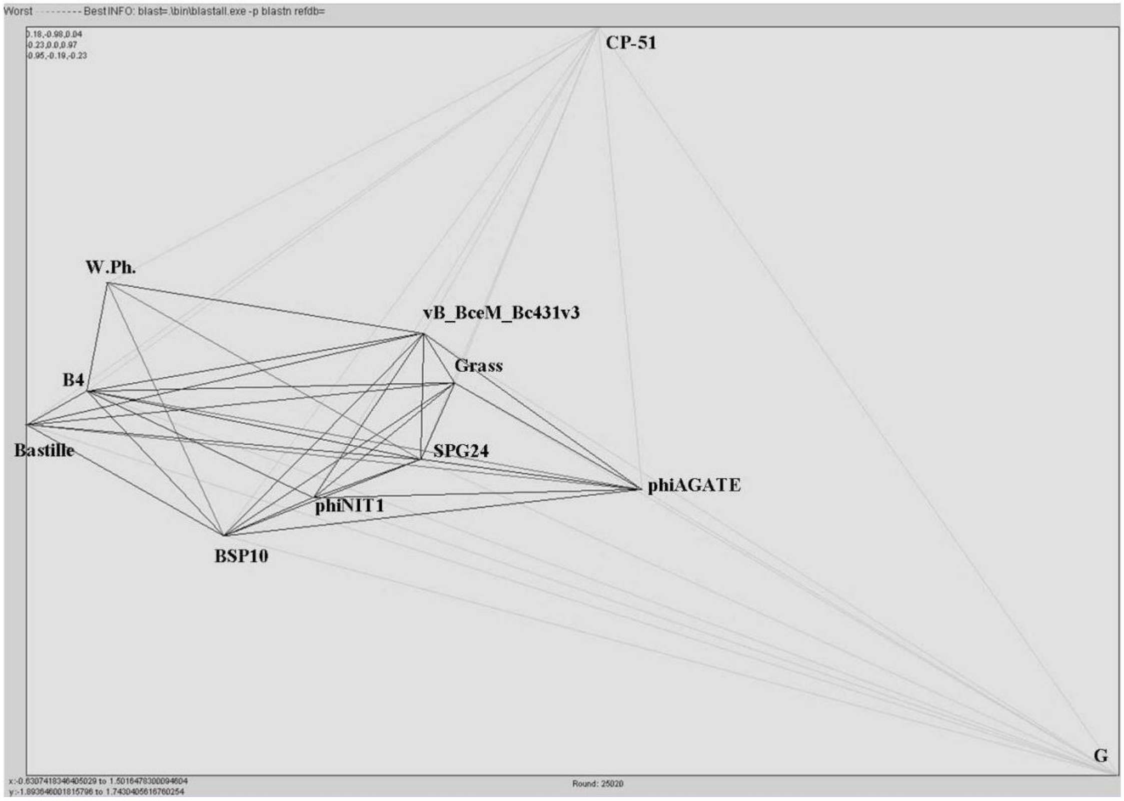

Figure S1. CLANS analysis of a total of 10 ICTV (International Committee on Taxonomy of Viruses)-classified *Bacillus* bacteriophages (phiNIT1, Grass, SPG24, phiAGATE, B4, Bastille, vB_BceM_Bc431v3, CP-51, W.Ph., and G), and bacteriophage BSP10. Edge weights were calculated from the P values of the BLASTN high-scoring segment pairs (e-value cut-off =1e-5). The network was visualized after 25000 runs using the CLANS software package.

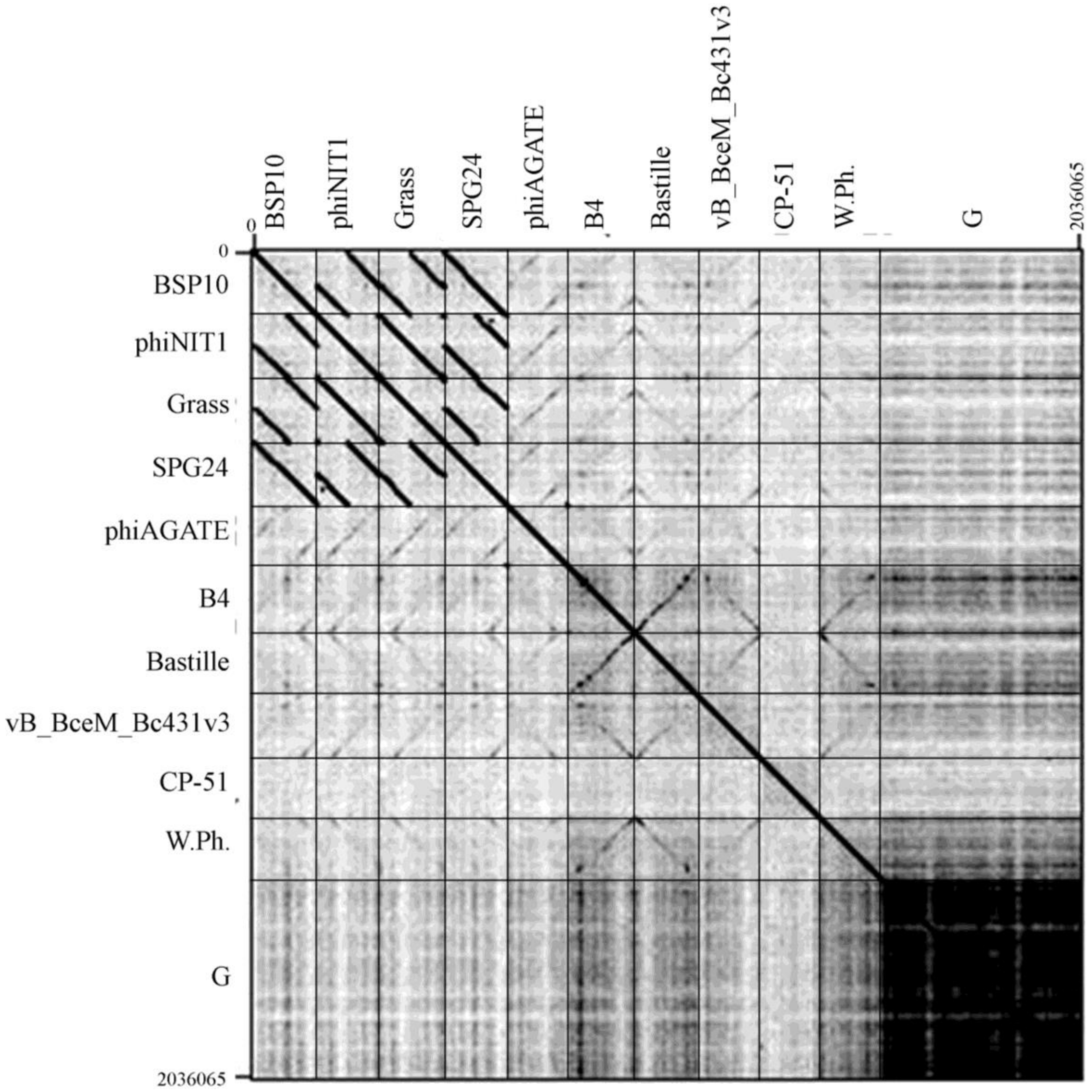

Figure S2. Whole-genome nucleotide dot plot of bacteriophage BSP10 and other ICTV (International Committee on Taxonomy of Viruses)-classified *Bacillus* bacteriophage (phiNIT1, Grass, SPG24, phiAGATE, B4, Bastille, vB_BceM_Bc431v3, CP-51, W.Ph., and G). Dot plots of whole genome sequences were generated using Gepard program.

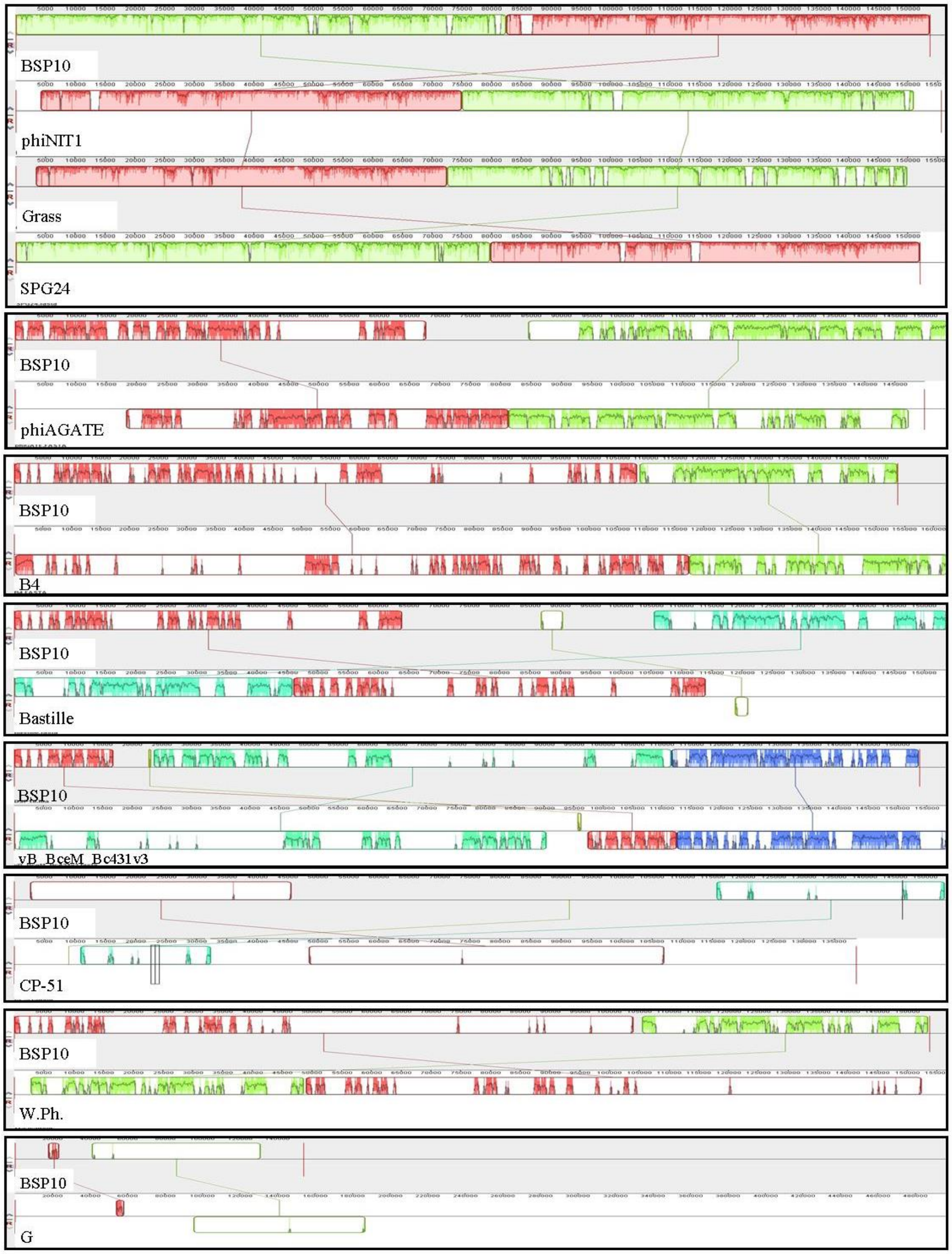

Figure S3. Bacteriophage BSP10 genome is compared with genus *Nit1virus* (bacteriophage phiNIT1, Grass, and SPG24), *Agatevirus* (phiAGATE), *B4virus* (B4), *Bastillevirus* (Bastille), *Bc431virus* (vB_BceM_Bc431v3), *Cp51virus* (CP-51), and *Wphvirus* (W.Ph.) as well as unassigned phage G using progressiveMauve alignment. Colored blocks indicate 1 to 1 best alignment and the sequence similarity is indicated by similarity plot within colored blocks.

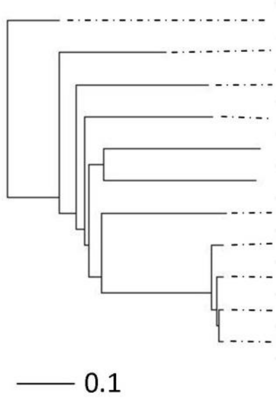

	1	2	3	4	5	6	7	8	9	10	11
1: B4	100,0	62,1	54,7	56,0	46,3	49,4	51,9	52,4	52,2	52,2	52,4
2: Bastille	63,2	100,0	56,3	55,0	46,2	49,3	51,3	51,9	51,7	51,7	51,7
3: W.Ph.	54,9	56,0	100,0	52,1	46,2	49,8	49,7	50,4	50,3	50,0	50,2
4: Bc431v3	56,2	54,5	52,0	100,0	46,2	49,1	52,5	54,0	53,7	53,6	53,8
5: CP-51	46,5	46,1	46,2	46,3	100,0	48,4	45,9	46,2	46,1	46,1	46,0
6: G	46,9	46,5	47,1	46,6	45,8	100,0	46,1	46,0	46,0	45,9	46,0
7: phiAGATE	52,7	51,5	50,2	53,4	46,1	49,2	100,0	57,7	57,5	57,5	57,7
8: Grass	52,5	51,5	50,3	54,1	46,0	48,7	56,9	100,0	95,8	95,6	95,6
9: phiNIT1	52,4	51,5	50,3	53,9	45,9	48,7	56,8	96,0	100,0	97,9	97,6
10: SPG24	52,6	51,5	50,1	53,9	45,9	48,7	57,2	96,0	97,8	100,0	98,4
11: BSP10	52,6	51,5	50,2	54,0	45,9	48,7	57,2	95,9	97,6	98,5	100,0

Figure S4. Heat-plot showing the results of genome comparison of bacteriophage BSP10 with other ICTV-classified *Bacillus* phage and the resulting phylogenetic tree. The similarity values were calculated using Gegenees software based on pairwise translated comparison of the analyzed sequences (tBLASTx method, fragment size –50, step size –25, threshold 10%). The heat plot colors reflect this similarity, ranging from low (red) to high (green). The tree was constructed with SplitsTree using the neighbor-joining method. The scale bar represents a 10% difference in the average tBLASTx score.
