# Supplementary material for: Complete Nucleotide Sequence Analysis of a Novel Bacillus subtilis-Infecting Bacteriophage BSP10 and Its Effect on Poly-Gamma-Glutamic Acid Degradation
Source: Viruses. 2018 May 4;10(5):240. doi: 10.3390/v10050240 (PMC5977233; doi:10.3390/v10050240)
Supplement: Supplementary file 1 [file viruses-10-00240-s001.zip › Supplementary Tables.pdf]

Supplementary Tables

Table S1. Sensitivity of *Bacillus subtilis* strains against *Bacillus* bacteriophage BSP10.

Bacteria name	Bacteriophage BSP10	Bacteria name	Bacteriophage BSP10
Bacillus subtilis ATCC 19659	-	*Bacillus subtilis* KCCM 12511	+
Bacillus subtilis ATCC 31028	-	*Bacillus subtilis* KCCM 12512	+
Bacillus subtilis ATCC 21697	-	*Bacillus subtilis* KCCM 12513	+
Bacillus subtilis ATCC 15245	+	*Bacillus subtilis* KCCM 40084	-
Bacillus subtilis ATCC 21556	-	*Bacillus subtilis* KCCM 40820	+
Bacillus subtilis ATCC 35854	-	*Bacillus subtilis* KCCM 40821	-
Bacillus subtilis ATCC 21332	-	*Bacillus subtilis* KCCM 41462	+
Bacillus subtilis ATCC 15841	+	*Bacillus subtilis* KCCM 40088	-
Bacillus subtilis ATCC 6051	-	*Bacillus subtilis* KCCM 41990	+
Bacillus subtilis ATCC 14593	-	*Bacillus subtilis* KCCM 41991	+
Bacillus subtilis ATCC 33677	-	*Bacillus subtilis* KCCM 41992	+
Bacillus subtilis ATCC 21336	+	*Bacillus subtilis* KCCM 12247	-
Bacillus subtilis ATCC 21770	-	*Bacillus subtilis* KCCM 11780	-
Bacillus subtilis ATCC 29233	-	*Bacillus subtilis* KCCM 12248	-
Bacillus subtilis ATCC 21228	+	*Bacillus subtilis* KCCM 11314	-
Bacillus subtilis ATCC 23059	+	*Bacillus subtilis* KCCM 11732	-
Bacillus subtilis ATCC 6633	-	*Bacillus subtilis* KCTC 2217	-
Bacillus subtilis KCCM 11496	-	*Bacillus subtilis* KACC 10114	-
Bacillus subtilis KCCM 11731	-	*Bacillus subtilis* KACC 10112	-
Bacillus subtilis KCCM 11733	-	*Bacillus subtilis* KACC 17802	+
Bacillus subtilis KCCM 11736	+	*Bacillus subtilis* KACC 17796	+
Bacillus subtilis KCCM 11738	-	*Bacillus subtilis* KACC 17797	+
Bacillus subtilis KCCM 12052	-	*Bacillus subtilis* SRCM 101274	-
Bacillus subtilis KCCM 12053	-	*Bacillus subtilis* SRCM 100170	-
Bacillus subtilis KCCM 12150	-	*Bacillus subtilis* SRCM 100336	-
Bacillus subtilis KCCM 12151	-	*Bacillus subtilis* SRCM 100169	-

+, clear lysis and forming plaques; -, no lysis

Table S2. Functional grouping of annotated ORFs in *Bacillus* bacteriophage BSP10.

Functional Group	ORF	Putative function	Similar ORF of other phages			
			Phage name	% Identity (BLASTP)	E-value	Protein Accession No.
Structure	ORF1	putative tail protein	phiNIT1	96	0	YP_008318362.1
	ORF3	minor capsid protein	SPG24	80	7.00E-114	YP_009273292.1
	ORF206	putative prohead protease	phiNIT1	99	0	YP_008318326.1
	ORF208	putative major capsid protein	phiNIT1	99	0	YP_008318328.1
	ORF215	tail sheath protein	Grass	99	0	YP_008771442.1
	ORF223	putative tail fiber 1	Grass	99	0	YP_008771450.1
	ORF224	putative tail fiber 2	phiNIT1	98	0	YP_008318348.1
	ORF225	putative minor structural protein	phiNIT1	95	0	YP_008318350.1
	ORF231	baseplate assembly protein	Grass	98	2.00E-177	YP_008771458.1
	ORF232	putative baseplate protein 1	phiNIT1	99	0	YP_008318357.1
	ORF233	putative baseplate protein 2	Grass	98	0	YP_008771460.1
DNA replication, transcription, and repair	ORF4	putative intein containing DNA helicase	phiNIT1	99	0	YP_008318365.1
	ORF6	putative DNA helicase 1	phiNIT1	100	0	YP_008318367.1
	ORF7	putative DNA repair exonuclease 1	phiNIT1	100	0	YP_008318368.1
	ORF8	putative DNA repair exonuclease 2	phiNIT1	99	0	YP_008318369.1
	ORF10	putative primase	phiNIT1	98	0	YP_008318371.1
	ORF11	dUTPase	SPG24	99	0	YP_009273307.1
	ORF12	putative MarR family transcriptional regulator	phiNIT1	98	4.00E-70	YP_008318373.1
	ORF15	putative resolvase	phiNIT1	100	4.00E-133	YP_008318375.1
	ORF27	putative DNA binding protein 1	Grass	100	6.00E-62	YP_008771493.1
	ORF29	putative DNA polymerase	phiNIT1	99	0	YP_008318389.1
	ORF31	DNA polymerase C-terminal	phiNIT1	99	2.00E-161	YP_008318392.1
	ORF35	RecA	Grass	98	0	YP_008771502.1
	ORF37	RNA polymerase sigma factor 1	Grass	100	7.00E-152	YP_008771504.1
	ORF42	metallophosphatase	phiNIT1	99	0	YP_008318401.1
	ORF54	putative transcription rho factor	phiNIT1	92	3.00E-127	YP_008318411.1
	ORF58	putative DNA binding protein 2	phiNIT1	98	4.00E-131	YP_008318415.1

	ORF69	RNA polymerase sigma factor 2	Grass	96	0	YP_008771532.1
	ORF104	putative transcriptional regulator	Grass	100	1.00E-48	YP_008771564.1
	ORF179	putative nucleotidyltransferase-like protein	phiNIT1	100	0	YP_008318299.1
	ORF198	putative XRE family transcriptional regulator 1	phiNIT1	98	1.00E-55	YP_008318319.1
	ORF200	putative XRE family transcriptional regulator 2	phiNIT1	99	3.00E-68	YP_008318321.1
Nucleotide metabolism	ORF16	putative ribonucleotide reductase stimulatory protein	phiNIT1	100	1.00E-86	YP_008318376.1
	ORF17	putative ribonucleotide-diphosphatereductase alpha sudunit N-terminal protein	phiNIT1	100	1.00E-175	YP_008318377.1
	ORF19	putative ribonucleotide-diphosphatereductase sudunit alpha C-terminal protein	phiNIT1	99	0	YP_008318378.1
	ORF22	ribonucleotide-diphosphate reductase beta subunit	SPG24	99	0	YP_009273321.1
	ORF176	dihydrofolate reductase	Grass	99	5.00E-120	YP_008771402.1
	ORF177	putative adenylate kinase and related kinases	phiNIT1	98	5.00E-140	YP_008318297.1
	ORF178	putative thymidylate synthase	phiNIT1	99	0	YP_008318298.1
DNA packaging	ORF189	terminase large subunit	1102phi1-3	98	0	AAS45252.1
	ORF205	putative portal protein	phiNIT1	99	0	YP_008318325.1
Introns and homing endonuclease	ORF18	intron associated endonuclease	SPG24	100	9.00E-175	YP_009273314.1
	ORF30	HNH homing endonuclease 1	Moonbeam	42	9.00E-44	YP_009151680.1
	ORF48	HNH homing endonuclease 2	BCP8-2	48	8.00E-77	YP_009149704.1
	ORF85	HNH homing endonuclease 3	Moonbeam	50	2.00E-68	YP_009151745.1
Host lysis	ORF38	putative holin protein	phiNIT1	100	3.00E-64	YP_008318398.1
	ORF185	endolysin	phiNIT1	98	0	YP_008318305.1
	ORF221	endo-beta-N-acetylglucosamidase	Grass	96	0	YP_008771448.1
	ORF222	putative tail lysin	Grass	95	0	YP_008771449.1
Others	ORF24	thioredoxin	SIOphi	94	9.00E-46	AGK86984.1
	ORF83	plasmid segregation protein	Grass	98	0	YP_008771547.1
	ORF86	putative peptidase	phiNIT1	84	1.00E-111	YP_008318432.1

	ORF91	FtsK/SpoIIIE protein	phiNIT1	98	0	YP_008318436.1
	ORF119	putative conserved lipoprotein	Grass	98	2.00E-90	YP_008771583.1
	ORF172	poly-gamma-glutamate hydrolase	BSNPO1	99	8.00E-153	AEI59296.1
	ORF183	phosphate starvation-inducible protein	Grass	99	2.00E-168	YP_008771410.1
	ORF217	putative cell wall binding protein	Grass	100	1.00E-137	YP_008771444.1

Table S**3.** Homing endonuclease in bacteriophage BSP10 genome predicted by HHpred program.

ORF	Target gene	Homolog	Homing endonuclease family	E value
ORF18	Ribonucleotide-diphosphatereductase alpha subunit	1MK0 catalytic domain of intron endonuclease I-TevI, E75A mutant *Enterobacteria* phage t4 sensu lato	GIY-YIG	4.90E-20
ORF30	DNA polymerase	1U3E DNA binding and cleavage by the HNH homing endonuclease I-HmuI *Bacillus* virus SPO1	HNH	7.80E-09
ORF48	-	1U3E DNA binding and cleavage by the HNH homing endonuclease I-HmuI *Bacillus* virus SPO1	HNH	3.50E-11
ORF85	-	1U3E DNA binding and cleavage by the HNH homing endonuclease I-HmuI *Bacillus* virus SPO1	HNH	5.30E-32
